# A Letter to Dr. Simmons, F. R. S. from Mr. James Lucas, One of the Surgeons of the General Infirmary at Leeds

**Published:** 1787

**Authors:** James Lucas

**Affiliations:** Leeds


### VI. *A Letter to Dr.* Simmons, *F. R. S. from Mr.* James Lucas, *one of the Surgeons of the General Infirmary at Leeds.*

*To Dr.* Simmons.

SIR,

I AM ſorry to find that my obſervations upon amputation appear to have been, in ſome degree, liable to miſconſtruction*. An *early deciſion* upon the neceſſity of amputation, in caſes of accident, is often neceſſary, becauſe it would be injudicious to operate after the limb becomes inflamed; and the death of the patient has been known to happen before the ſymptomatic fever had ſufficiently abated: yet I do not find that "precipitate amputation" is, in general, preferred to the employment of ſuch means as are likely to preſerve the limb, or that "an operation is often neceſſary at the "end of a month or ſix weeks." My obſervation, on the increaſe of blood veſſels, is founded on experience. The "limitations" ſtated are not ſtrictly requiſite: the prognoſtic is not ſo difficult as ſeems to have been con-

* See Vol. VII. p. 377.

ceived; ceived; nor are "care and attention to take "up the veffels" *always* fufficient to reftrain the hæmorrhage *.

As it was not the *tibia*, but the *os femoris*, that was found protruded through the integuments, "an unguarded feparation of them "from the bone" is not eafy to be comprehended †.

My paper was intended not merely "to re- "vive the flap operation," but to point out an occafional preference in the different modes of amputation, and to enforce an exact attention to every part of the treatment, as being the only means of infuring fuccefs.

By having the apparatus completely provided, and the parts for incifion previoufly marked out, the operator is at liberty to attend more fully to the executive part; and though I have not advanced that fuch "minutiæ" are neceffary, yet I beg leave to repeat, that they have been found of material advantage ‡.

Although I have enlarged on unfavourable cafes only, yet I can, with equal truth, add, that

* Vol. VII. p. 383.

† Ibid. p. 387.

that no inſtance of failure has occurred in thoſe patients on whom amputations have been performed early; either in reſpect to their recovery, or the ready healing of their wounds: I am, therefore, inclined to think, that the want of ſucceſs, ſo alluded to, ſhould be rather attributed to ſome other cauſe, than "to the "healthy ſtate of the patient *."

The obſervations, which ſeem to have been ſomewhat haſtily criticiſed, were the reſult of twenty years experience, as an hoſpital ſurgeon, and had been frequently and carefully reconſidered: they were drawn up without a wiſh to ſupport any theory, but with a deſire to contribute my endeavours towards the eſtabliſhment of an improvement in ſurgery, which had repeatedly afforded me the greateſt ſatiſfaction.

I have the honour to remain,

SIR,

You moſt obedient Servant,

JAMES LUCAS.

*Leeds,*

April 21ſt, 1787.

* Vol. VII. p. 382, 390.

VII. *Some*

